# Supplementary material for: Novel Salinomycin-Based Paramagnetic Complexes—First Evaluation of Their Potential Theranostic Properties
Source: Pharmaceutics. 2022 Oct 28;14(11):2319. doi: 10.3390/pharmaceutics14112319 (PMC9692412; doi:10.3390/pharmaceutics14112319)
Supplement: Supplementary file 1 [file pharmaceutics-14-02319-s001.zip › pharmaceutics-1974263-supplementary.pdf]

Article

Novel Salinomycin-Based Paramagnetic Complexes—First Evaluation of Their Potential Theranostic Properties

Irena Pashkunova-Martic [1,*], Rositsa Kukeva [2], Radostina Stoyanova [2], Ivayla Pantcheva [3], Peter Dorkov [4], Joachim Friske [1], Michaela Hejl [5], Michael Jakupec [5], Mariam Hohagen [6], Anton Legin [5], Werner Lubitz [7], Bernhard K. Keppler [5], Thomas H. Helbich [1] and Juliana Ivanova [8]

[1] Department of Biomedical Imaging and Image-Guided Therapy, Division of Molecular and Structural
Preclinical Imaging, Preclinical Imaging Laboratory, Medical University of Vienna & General Hospital of Vienna, Waehringer Guertel 18–20, 1090 Vienna, Austria

[2] Institute of General and Inorganic Chemistry, Bulgarian Academy of Sciences, Akad. G. Bonchev Str., bl. 11, 1113 Sofia, Bulgaria

[3] Faculty of Chemistry and Pharmacy, Sofia University "St. Kliment Ohridski", J. Bourchier Blvd., 1, 1164 Sofia, Bulgaria

[4] Chemistry Department, R&D, BIOVET Ltd., 39 Peter Rakov Str., 4550 Peshtera, Bulgaria

[5] Institute of Inorganic Chemistry, University of Vienna, Waehringer Strasse 42, 1090 Vienna, Austria

[6] Department of Inorganic Chemistry—Functional Materials, University of Vienna, Waehringer Strasse 42, 1090 Vienna, Austria

[7] BIRD-C GmbH, Dr. Bohrgasse 2–8, 1030 Vienna, Austria

[8] Faculty of Medicine, Sofia University "St. Kliment Ohridski", Kozjak Str., 1, 1407 Sofia, Bulgaria

* Correspondence: irena.pashkunova-martic@meduniwien.ac.at; Tel.: +43-1-40400-48195; Fax: +43-1-40400-48980

Section S1. ESI-MS spectra and fragmentation scheme of $[Gd(C_{42}H_{69}O_{11})_3(H_2O)_3]$ complex:

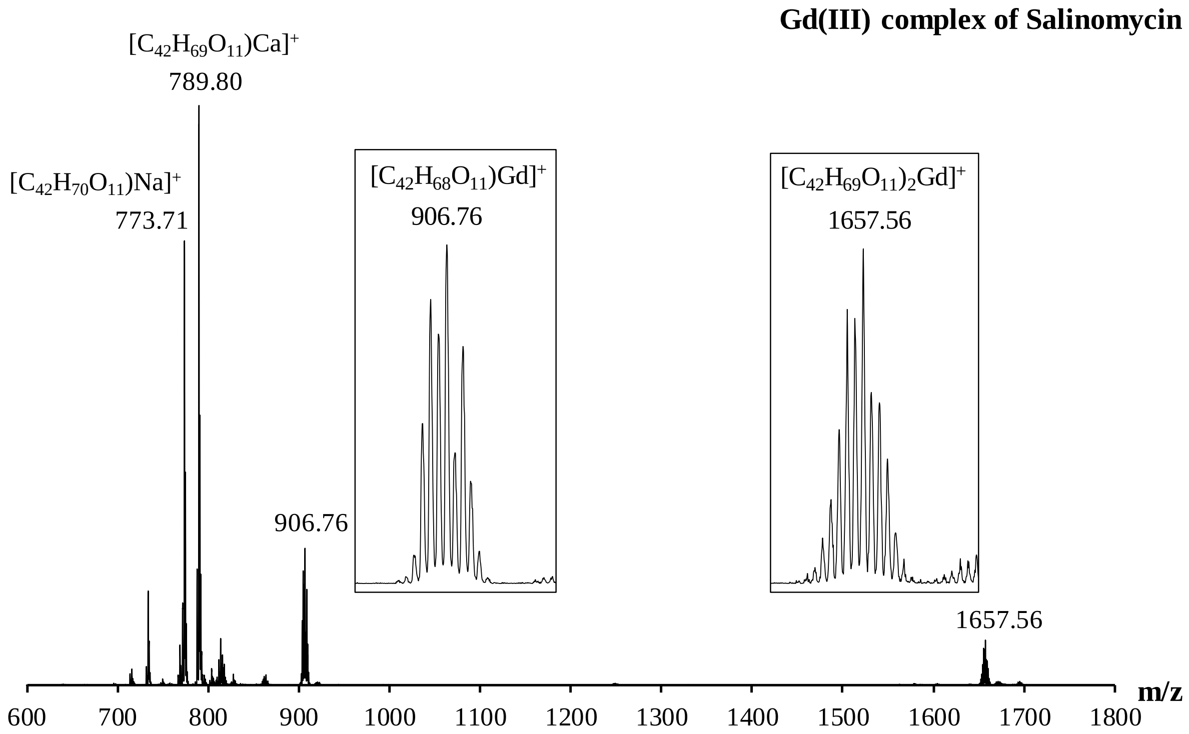

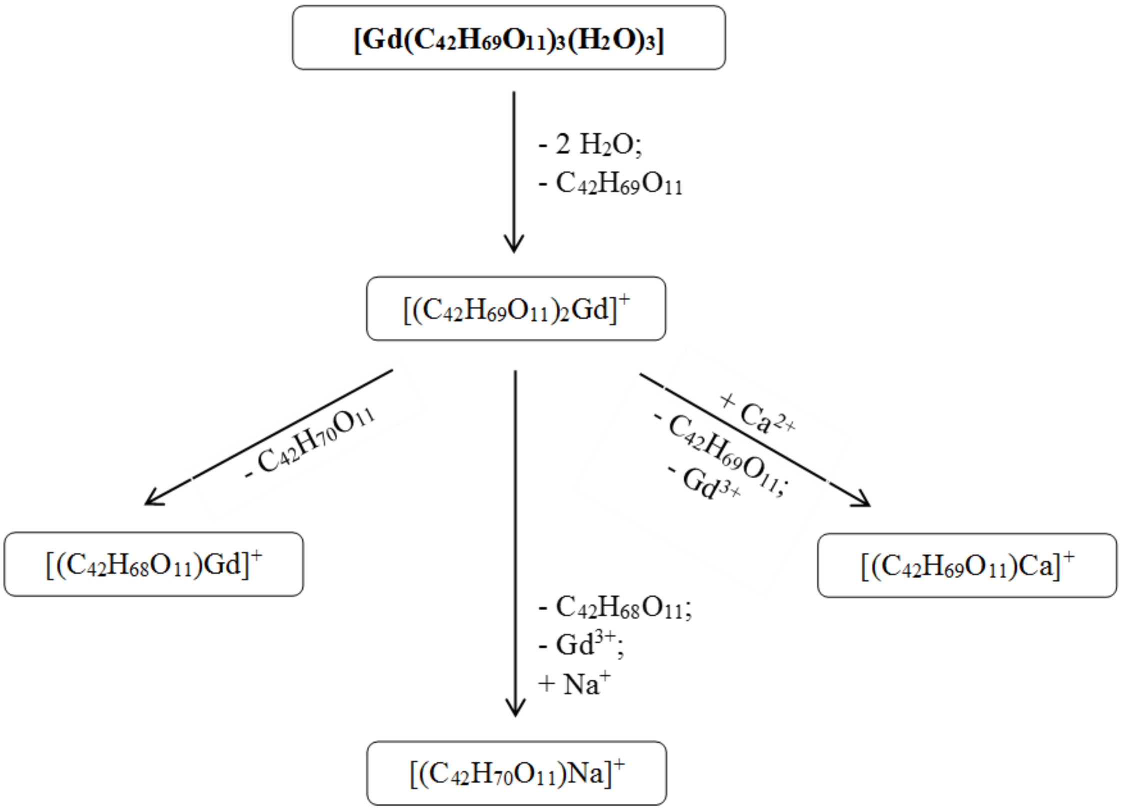

Section S2. ESI-MS spectra and fragmentation scheme of $[Mn(C_{42}H_{69}O_{11})_2(H_2O)_2]$ complex:

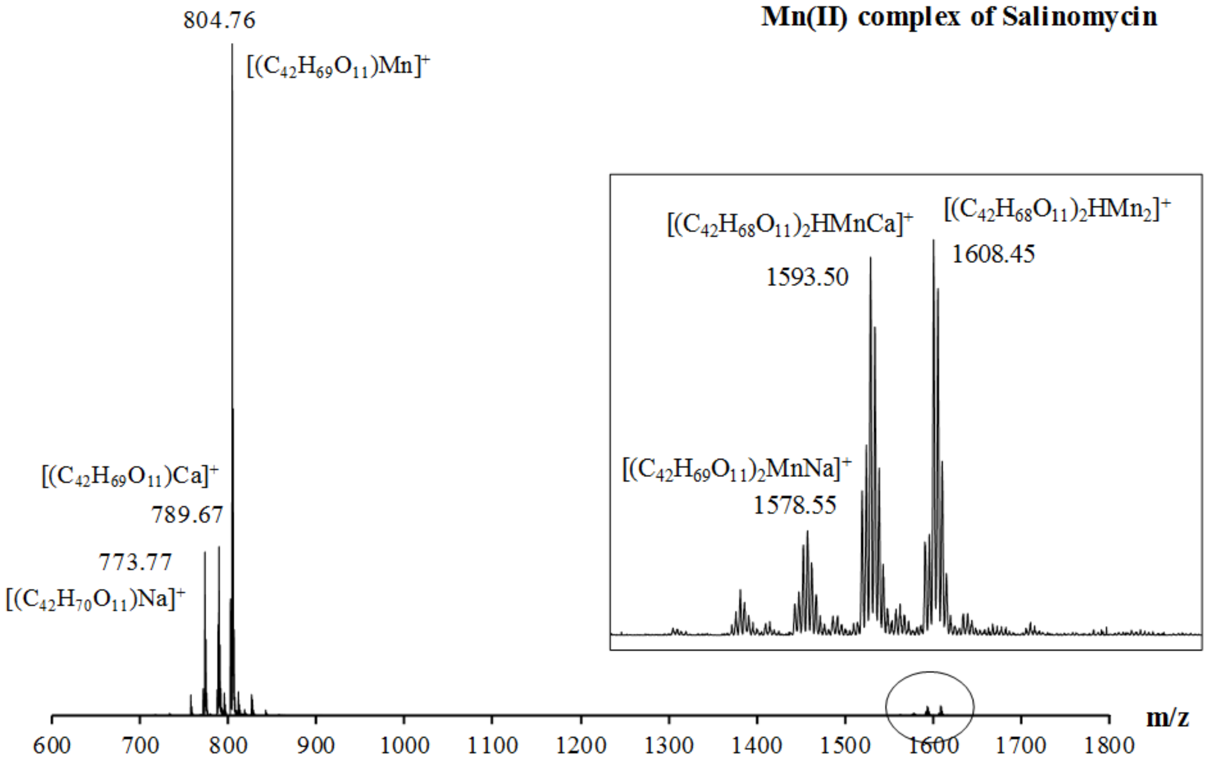

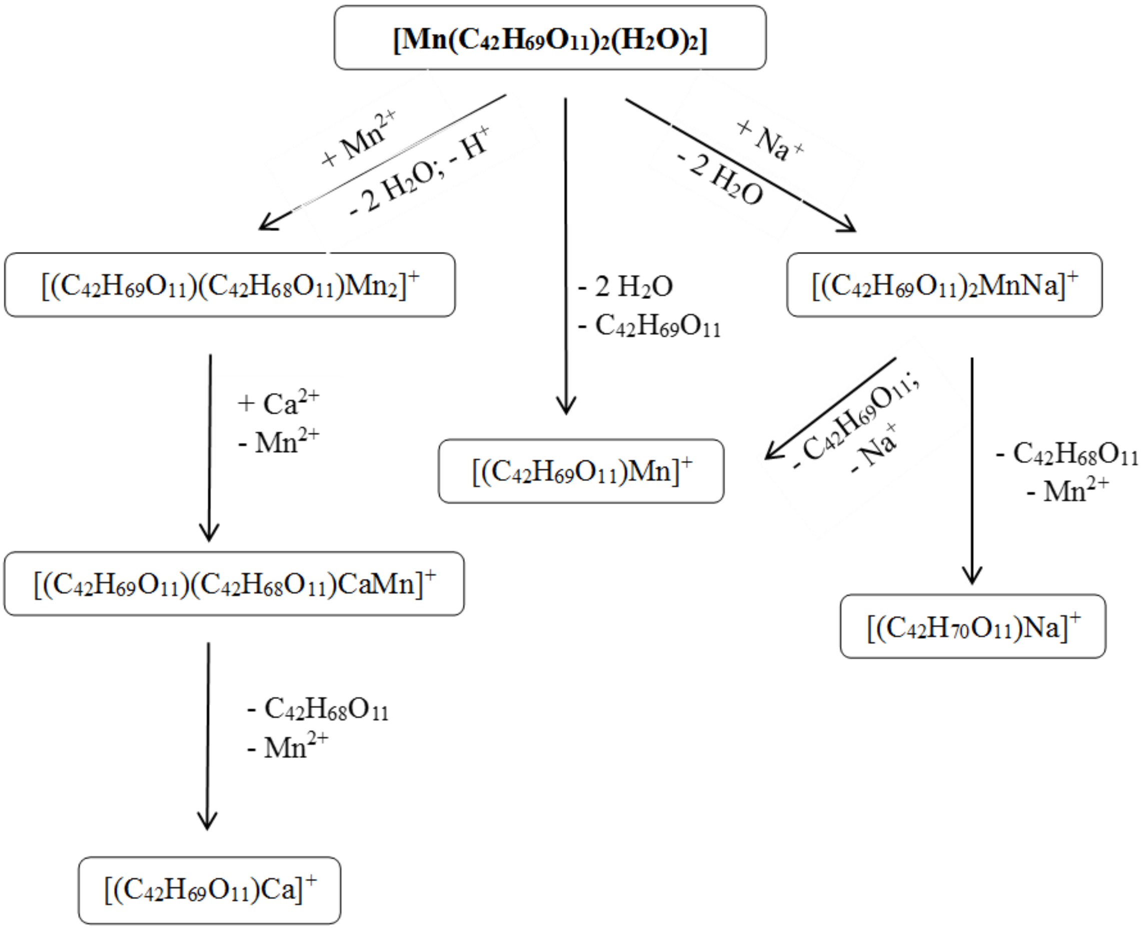

Section S3. IR spectra of SalH, Sal-Gd(III) Sal-Mn(II)

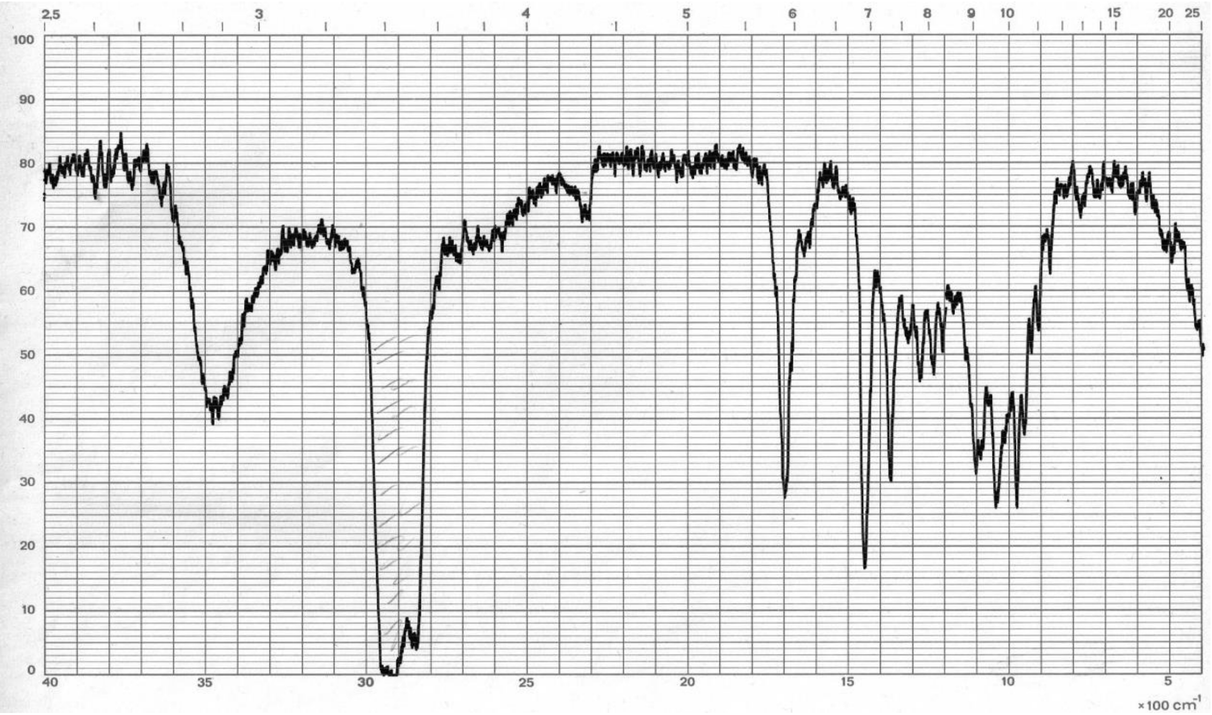

SalH

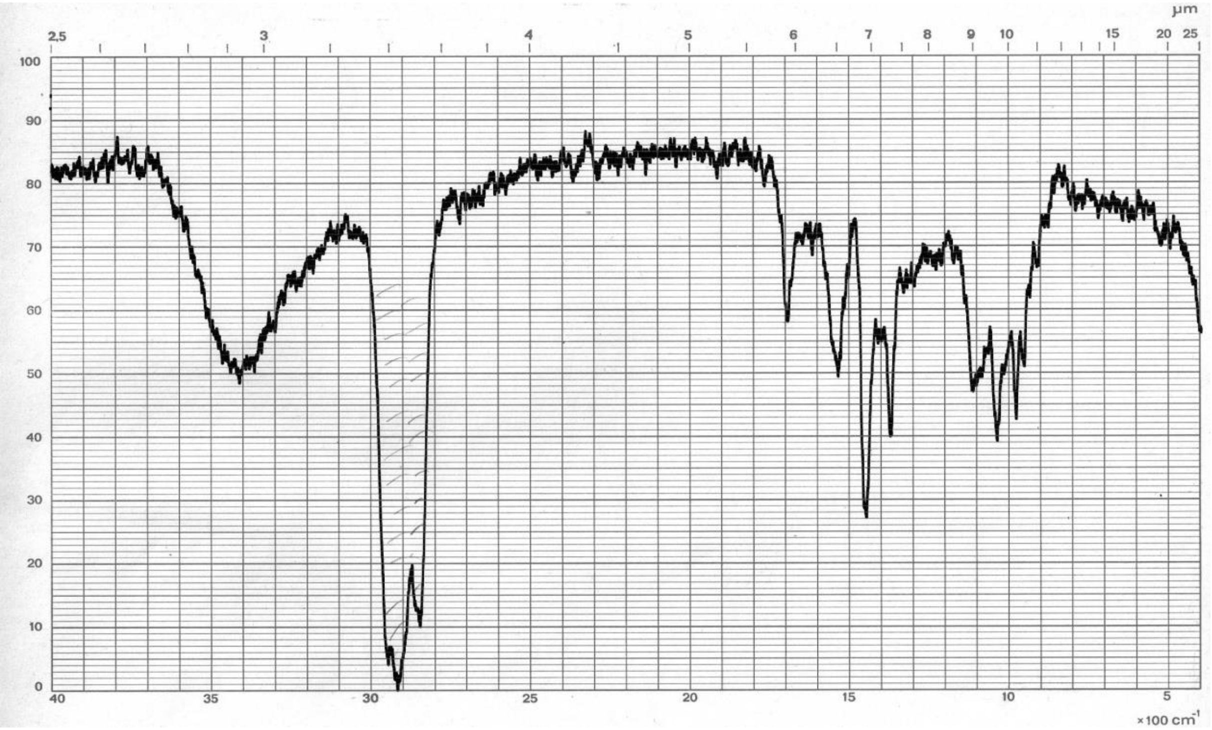

Sal-Gd(III)

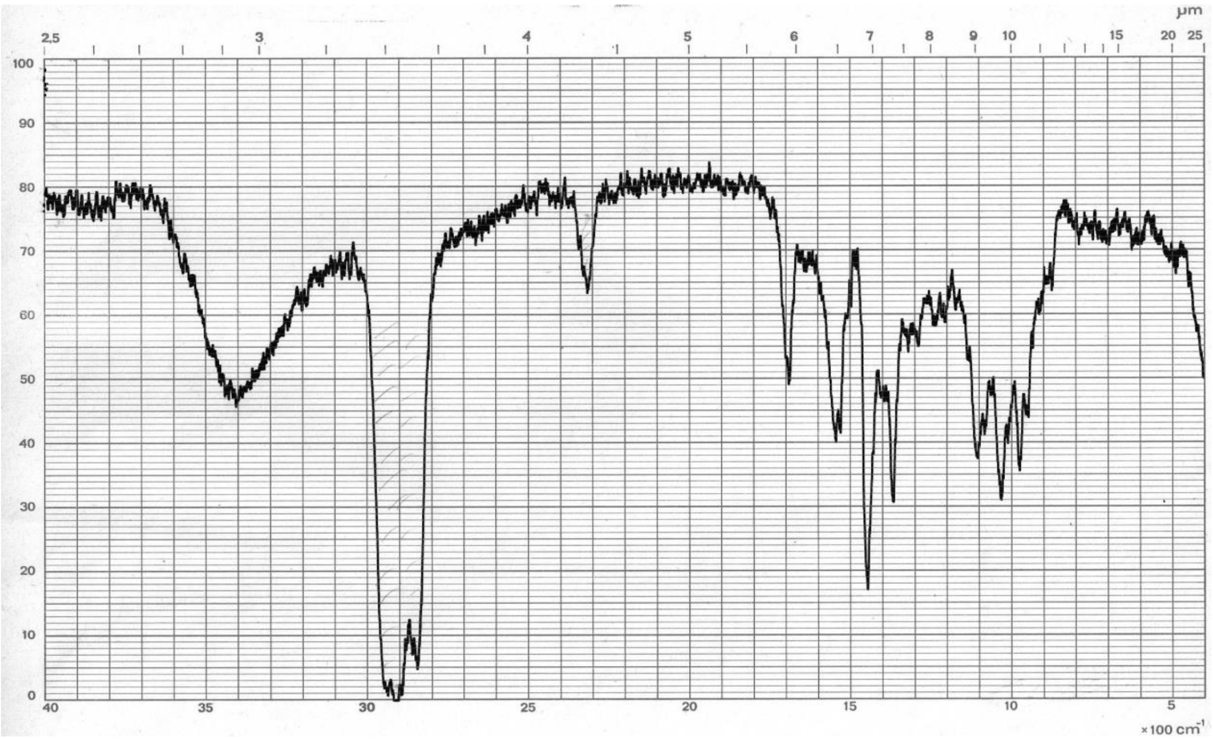

µm
2,5
3
4
5
6
7
8
9
10
15
20
25
100
90
80
70
60
50
40
30
20
10
0
40
35
30
25
20
15
10
5
×100 cm⁻¹

Sal-Mn(II)

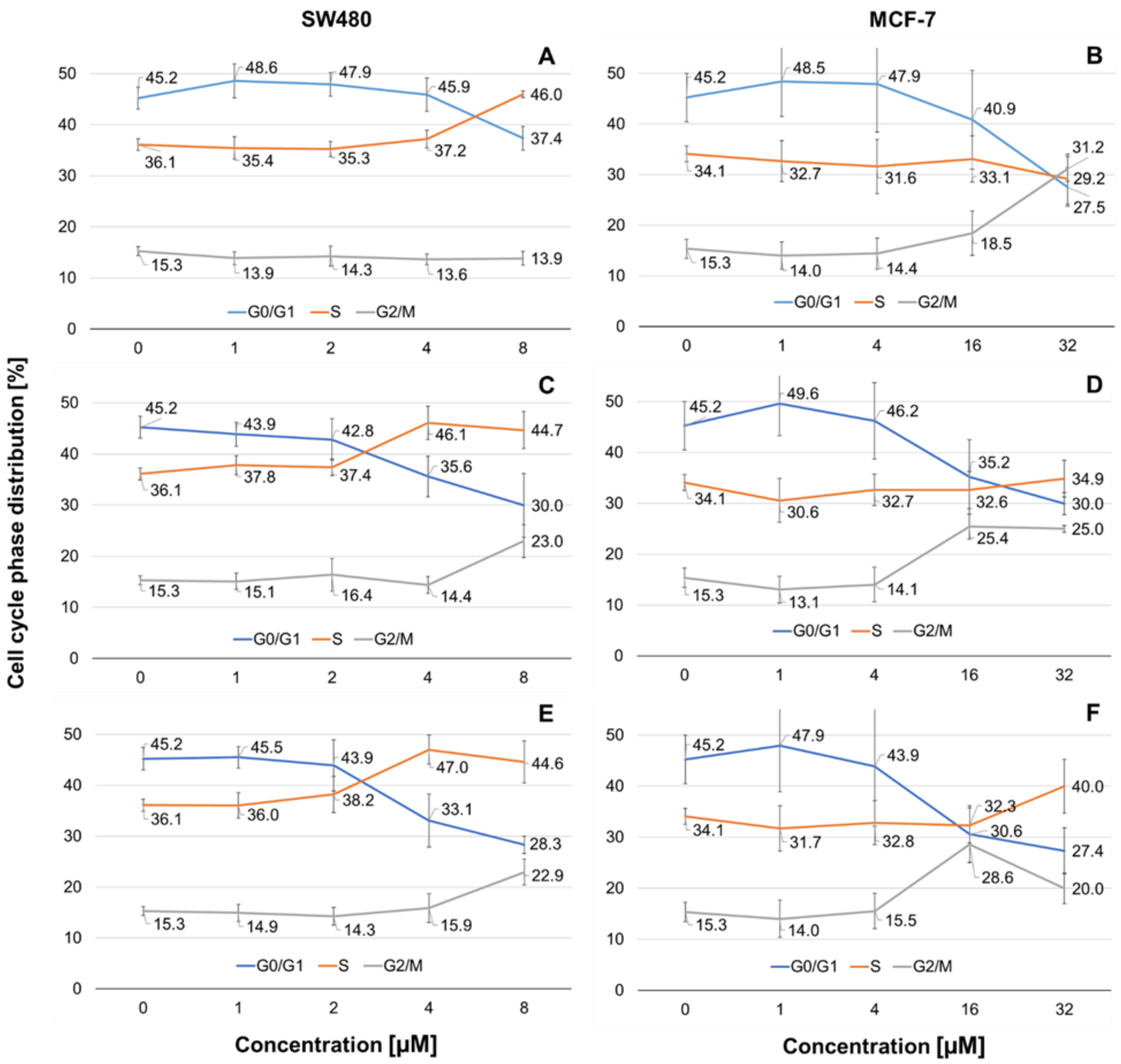

Figure S1. Cell cycle alterations in SW480 (left) and MCF-7 (right) cancer cells. The cell cultures were exposed to increasing concentrations of Sal-H (**A**, **B**), Sal-Mn(II) (**C**, **D**) and Sal-Gd(III) (**E**, **F**) for 24 h.

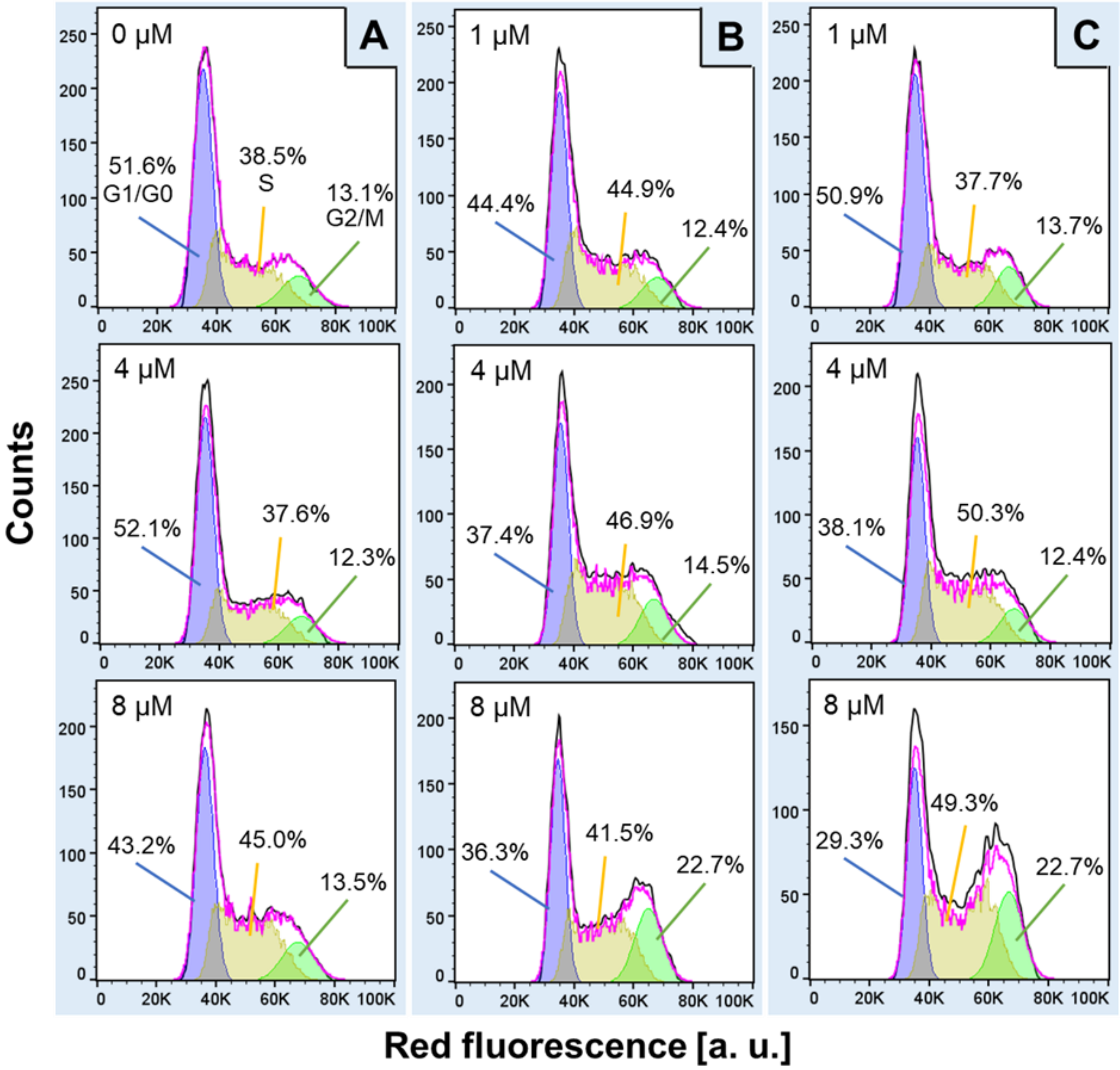

Figure S2. Histograms of cell cycle phase distribution in SW480 cells exposed to increasing concentrations of Sal-H (column **A**), Sal-Mn(II) (column **B**) and Sal-Gd(III) (column **C**) for 24 h. Top left histogram represents an untreated control.

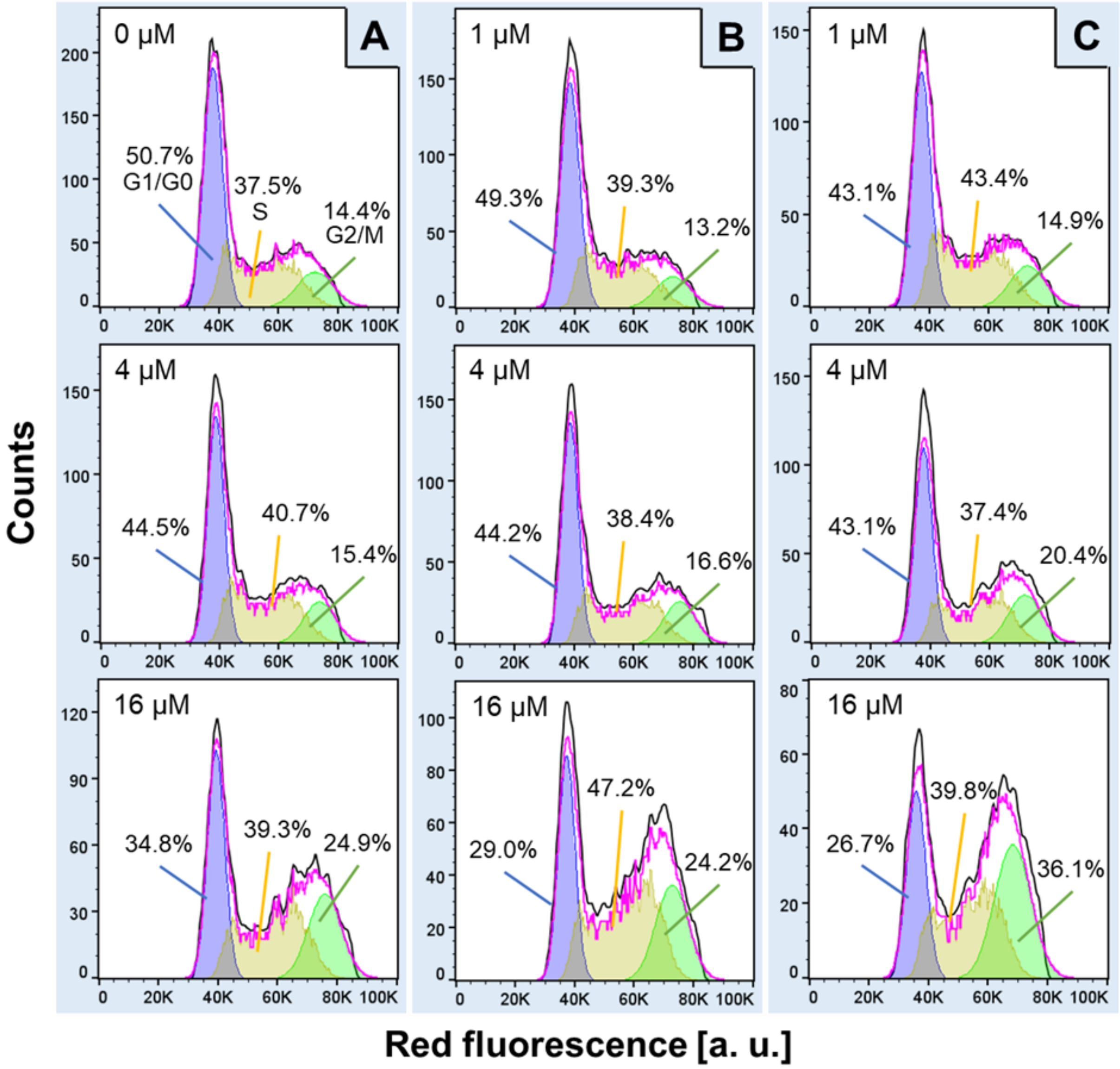

Figure S3. Histograms of cell cycle phase distribution in MCF-7 cells exposed to increasing concentrations of Sal-H (column **A**), Sal-Mn(II) (column **B**) and Sal-Gd(III) (column **C**) for 24 h. Top left histogram represents an untreated control.
